# *Coxiella burnetii* Infections Identified by Molecular Methods, United States, 2006–2023

**DOI:** 10.3201/eid3104.241214

**Published:** 2025-04

**Authors:** Caroline K. Maki, Thao T. Truong, Johanna S. Salzer, Nicolette Bestul, Brad T. Cookson, Gilbert J. Kersh, Stephen J. Salipante, Joshua A. Lieberman, David W. McCormick

**Affiliations:** Centers for Disease Control and Prevention, Atlanta, Georgia, USA (C.K. Maki, J.S. Salzer, N. Bestul, G.J. Kersh, D.W. McCormick); Oak Ridge Institute for Science and Education, Oak Ridge, Tennessee, USA (C.K. Maki); University of Washington, Seattle, Washington, USA (T.T. Truong, B.T. Cookson, S.J. Salipante, J.A. Lieberman)

**Keywords:** Q fever, *Coxiella burnetii*, bacteria, surveillance, endocarditis, polymerase chain reaction, PCR, zoonoses, United States

## Abstract

We identified 34 patients with *Coxiella burnetii* infection using PCR; 31 (86%) cases were diagnosed from cardiac specimens. Nearly half (15/31, 48%) of those cases were not reported to any channel of national disease surveillance, indicating substantial underreporting for diseases identified using molecular methods at noncommercial laboratories.

Q fever, a nationally notifiable disease caused by *Coxiella burnetii* bacteria, has acute or chronic manifestations in humans (*1*). In accordance with the Council for State and Territorial Epidemiologists case definition published in 2008 (*2*), confirmed cases must meet clinical criteria and have either confirmatory laboratory evidence via paired serology or molecular methods, including PCR, or a confirmed epidemiologic link (*3*). Probable cases are those with clinically compatible symptoms and presumptive laboratory evidence (*3*). Suspected cases are those reported to local and state health departments by clinicians and laboratories where health department staff classify cases as confirmed or probable and report to US Centers for Disease Control and Prevention through the National Notifiable Diseases Surveillance System (NNDSS) using a standardized case report form (CRF). During 2008–2019, an annual average of 131 acute and 30 chronic cases were reported to CDC (*4*). Although PCR is a useful diagnostic modality for Q fever, it is not clear if cases identified using molecular methods at noncommercial laboratories are captured in national surveillance data (*3*,*5*).

We searched the laboratory information system for specimens submitted to the University of Washington Medicine Molecular Microbiology clinical diagnostic reference laboratory (UWMMD) with *C. burnetii* identified by broad-range bacterial PCR assay (*6*) or 16S amplicon next-generation sequencing (*7*). Acceptable specimen types were fresh-frozen tissue, body fluids other than blood, and formalin-fixed paraffin-embedded tissue. We obtained information on specimen type, patient age and sex, date of specimen collection, and submission state for 35 specimens from 34 patients. We attempted to match those patients to CRFs reported to CDC (*3*) by using patient age at time of specimen collection, sex, test result date, state from which the specimen was submitted, and specimen type information. We also matched using NNDSS data based on age and state of residence; those matches were not considered as strong as the CRF matching because NNDSS data do not include laboratory results. We performed descriptive statistical computations using R Studio 2023.06.1 (Posit, http://www.rstudio.com). The University of Washington Institutional Review Board approved the study (IRB; approval no. STUDY00013877); the study was exempt from IRB requirements, in accordance with CDC policies and procedures.

Median age of 34 identified patients was 62.5 (range 23–79) years; 19 (56%) were male and 15 (44%) were female (Table 1). The most common states for specimen collection were California (9 [27%]), Washington (7 [21%]), and Oregon (2 [6%]). Most (30/35 [86%]) specimens were cardiac tissue involving the aortic or mitral valves (23/30 [77%]). Eight (27%) specimens were prosthetic valves.

**Table 1:** Characteristics of case-patients with invasive *Coxiella burnetii* infection identified by molecular methods, United States, 2006–2023*

Characteristic	No. (%)
Sex	
M	19 (56)
F	9 (27)
NA	6 (18)
State of residence	
California	9 (27)
Washington	7 (21)
Ohio	6 (18)
Utah	4 (12)
Texas	2 (6)
Oregon	2 (6)
Montana	1 (3)
Virginia	1 (3)
Kentucky	1 (3)
Nevada	1 (3)
Specimen type, n = 35†	
Cardiac	30 (86)
Aortic‡	18 (78)
Mitral	5 (22)
Pulmonary	1 (50)
Tricuspid	1 (50)
Heart NOS§	5 (17)
Not cardiac	5 (14)
Synovial fluid	1 (2)
Knee joint	1 (2)
Chest cyst fluid	1 (2)
Psoas abscess	1 (2)
Retrocaval abscess	1 (2)

*Median age of patients at sample collection was 62.5 years; age range 23–79 years. NA, not available; NOS, not otherwise specified. †One patient had 2 positive specimens (tricuspid and mitral valve). ‡Six aortic specimens were prosthetic valves. §Two heart NOS specimens were prosthetic valves.

Matching was not possible for 3 patients because surveillance data from 2023 were not submitted to CDC at the time of this investigation. Of the 16 (52%) patients reported in surveillance data, 3 (10%) had data via CRF alone, 8 (26%) had data via NNDSS alone, and 5 (16%) had data through both sources (Table 2). Among 31 patients with invasive *C. burnetii* infections identified during 2006–2023 using molecular methods, 48% had no surveillance data submitted to either reporting channel. That finding suggests that Q fever cases identified using molecular methods are not adequately captured in routine public health surveillance. Previous studies have detailed substantial underreporting of both acute and chronic Q fever in the United States (*8*,*9*). The lack of reporting of nearly half of the tissue invasive infections we identified using molecular methods is especially concerning because invasive cases are likely to be more severe (*8*).

**Table 2:** Invasive *Coxiella burnetii* infection identified by molecular methods and matched to surveillance data, United States, 2006–2023*

State of residence	Total UW cases	Matched to surveillance data, no. (%), n = 31			
		Matched to CRF	Matched to NNDSS	Matched to both	Matched to either
California	9	1 (33)	2 (22)	2 (22)	5 (55)
Washington	7	1 (14)	2 (29)	0 (0)	3 (43)
Ohio	6	0 (0)	2 (33)	0 (0)	2 (33)
Utah	4	1 (25)	0 (0)	0 (0)	1 (25)
Texas	2	0 (0)	1 (50)	1 (50)	2 (100)
Oregon	2	0 (0)	1 (50)	0 (0)	1 (50)
Montana	1	0 (0)	0 (0)	0 (0)	0 (0)
Virginia	1	1 (100)	0 (0)	0 (0)	1 (100)
Kentucky	1	0 (0)	0 (0)	1 (100)	1 (100)
Nevada	1	0 (0)	0 (0)	0 (0)	0 (0)
Total	34†	3 (10)	8 (26)	5 (16)	16 (52)

*C. burnetii specimens tested positive at the University of Washington Molecular Microbiology clinical diagnostic reference laboratory were matched with surveillance data received through case report forms or the NNDSS. CRF, case report form; NNDSS, National Notifiable Diseases Surveillance System. †Matching was only feasible for 31 patients due to missing surveillance from states via CRF or unavailability of NNDSS data for 2023.

Testing laboratories are responsible for notifying local or state public health departments of positive cases. However, *C. burnetii* molecular testing on invasive tissue infections is typically performed at specialized referral laboratories that may be outside of the patient’s state of residence. Consistent with state policies that require reporting to the local public health jurisdiction, out-of-state referral laboratories do not routinely report back to the patient’s public health department; the clinician who ordered testing or the local clinic laboratory are typically required to report to the patient’s home state.

*C. burnetii* was identified in cardiac specimens among most (86%) patients in this cohort. Endocarditis accounts for 60%–78% of chronic Q fever cases globally (*3*), and patients with previous valvulopathy or with prosthetic valves are at increased risk (*2*,*10*). The higher proportion of endocarditis identified in this case series likely reflects referral practices from submitting institutions, particularly the common use of molecular testing in cases of suspected culture-negative endocarditis. We also observed bias in referral practices in the geographic skew to the West Coast, given UWMMD’s location in Washington.

In summary, our findings suggest substantial underreporting of *C. burnetii* cases identified through molecular methods as well as the need to create mechanisms that streamline reporting notifiable conditions from laboratories outside a patient’s home public health jurisdiction. Lack of clinician knowledge on reporting protocols, combined with miscommunication between healthcare providers, hospital laboratories, and state and local health departments, might contribute to the underreporting of *C. burnetii* infection we observed.
